# Factors influencing willingness to pay for healthcare

**DOI:** 10.1186/1471-2458-12-S2-A37

**Published:** 2012-11-27

**Authors:** Azimatun Noor Aizuddin, Saperi Sulong, Syed Mohamed Aljunid

**Affiliations:** 1 Department of Community Health, Universiti Kebangsaan Malaysia Medical Centre, Jalan Yaacob Latiff, 56000 Kuala Lumpur, Malaysia; 2 Department of Health Information, Universiti Kebangsaan Malaysia Medical Centre, Jalan Yaacob Latiff, 56000 Kuala Lumpur, Malaysia; 3 United Nations University - International Institute for Global Health, Universiti Kebangsaan Malaysia Medical Centre, Jalan Yaacob Latiff, 56000 Kuala Lumpur, Malaysia

## Background

Health financing is a sensitive issue that is currently being discussed around the world. Main concerns related to this are quality of health services and increasing healthcare cost. The most important features of health services that must be preserved are its equitability, affordability and quality. In many developing countries people are expected to contribute to the cost of healthcare from their own resources. The objective of this review is to identify the factors that influence willingness to pay for healthcare.

## Method

This is a review of various published and unpublished papers, studies and articles on the subject of willingness to pay for healthcare. Search engines used were Google, Yahoo, Science Direct & Medline and keywords entered includes ‘willingness to pay’, ‘health financing’ and ‘healthcare payment’. Studies of the years 1990 to 2011 were retrieved for the review.

## Results

Many studies showed that willingness to pay (WTP) can be influenced by few factors. The factors that were found to have significant associations with WTP were age, education, income, dependency ratio/ household size, perception, healthcare services quality, locality rural/ urban and ability to pay. However, there were also studies that found contrasting results. Other important factors that influence an individual’s WTP for health services are the marginal cost (the incremental price and level of utility) of a particular service or good and access to health services provided. Interestingly, price level does not influence WTP for health care.

## Conclusion

Willingness to pay for health care is beyond the financial capacity of a person and has multifactorial influences. It is recommended that governments review existing health services (public and private) and address all related matters prior to implementing a new healthcare financing system.

